# Association between *MUC1* gene polymorphism and expected progeny differences in Nelore cattle (*Bos primigenius indicus*)

**DOI:** 10.1590/S1415-47572009005000098

**Published:** 2010-03-01

**Authors:** Fabio Ricardo Pablos de Souza, Pedro Alejandro Vozzi, Reginaldo Aparecido Vila, Arione Augusti Boligon, Marli Aparecida Vani Galerani, Raysildo Barbosa Lobo, Lucia Regina Martelli

**Affiliations:** 1 Departamento de Genética, Faculdade de Medicina de Ribeirão Preto, Universidade de São Paulo, Ribeirão Preto, SPBrazil; 2 Departamento de Zootecnia, Faculdade de Ciências Agrárias e Veterinárias, Universidade Estadual Paulista Júlio de Mesquita Filho, Jaboticabal, SPBrazil

**Keywords:** beef cattle, molecular marker, productive traits, quantitative trait locus

## Abstract

MUC1 is a heavily glycosylated mammalian transmembrane protein expressed by mucosal secretory tissues for both protection against microbial infection and lubrication. An important characteristic of MUC1 is its variable number of tandem repeats (VNTR) containing several sites for O-glycosylation. VNTR length has been associated with many human diseases and with certain economically important traits in domestic ruminants. The aim of the present study was to correlate the length of MUC1 gene VNTR with expected progeny differences (EPDs) obtained for growth, fertility and carcass traits. Five alleles were identified, with alleles containing short VNTRs being more frequent than those with long, thereby demonstrating that Brazilian Nelore cattle are characterized by high frequencies in short MUC1 VNTRs. Statistical analyses revealed there to be no significant association between VNTR length and EPDs for weight at 120 days (W_120_ ), scrotal circumference at 365 (SC _365_ ) and 450 (SC _450_ ) days, age at first calving (AFC), and rib eye area (REA).

Most economically important traits in livestock are characterized by complex continuously distributed phenotypes, which are influenced by multiple genes located at quantitative trait loci (QTL) dispersed across the genome. Marked advances in production efficiency have been achieved following the implementation of sophisticated selection strategies based on quantitative genetics theory. One of the strong points inherent in these biometrical selection strategies is that they do not require a detailed understanding of the genes upon which they act. Moreover, there is great interest in gaining further knowledge on the molecular architecture of complex quantitative traits, this possibly leading to novel insights into the evolutionary forces to which natural and domestic populations are submitted, as well as the molecular physiology of phenotypes of interest. In addition, this knowledge is expected to generate new opportunities for more effective marker-assisted selection.

Transmembrane mucin 1 (MUC1) is a heavily glycosylated mammalian protein which is co-dominantly expressed on the apical surface of secretory tissues in mammary glands and the respiratory, gastrointestinal, urinary and reproductive tracts. The main functions of this protein are related to protection, lubrication, cell adhesion and cell signaling in response to extracellular changes in pH, salt concentration and osmolarity (Gendler, 2001).

The bovine MUC1 glycoprotein has been well characterized by sequencing of the corresponding bovine mammary gland cDNA. The main domains are a cytoplasmic C-terminal tail, a hydrophobic transmembrane domain, an extracellular domain containing a variable number of tandem repeats (VNTR), and the signal peptide. An important structural feature is the polymorphic VNTR which consists of repeats of 20-amino acid units rich in serine and threonine residues, being essential for O-glycosylation (Pallesen *et al.*, 2001).

In humans, VNTR polymorphism in this gene has been associated with conditions that precede gastric carcinoma (Carvalho *et al.*, 1997), severe acne (Ando *et al.*, 1998) and, in women, infertility due to failure in implantation (Horne *et al.*, 2001). In cattle of the taurine (*Bos primigenius taurus)* and zebuine (*Bos primigenius indicus*) breeds (Rasero *et al.*, 2002; Souza *et al.*, 2007; Hiendleder *et al.*, 2008), besides goats (*Capra hircus*) (Sacchi *et al.*, 2004) and sheep (*Ovis aries*) (Rasero *et al.*, 2007), the polymorphism has been studied in terms of usefulness in breeding programs. Various associations between MUC1 phenotype and milk-fat yield, milk-fat percentage and milk-protein yield have been reported in Holstein cattle (Hens *et al.*, 1995).

In Brazil, most beef-breeder associations supply information regarding expected progeny differences (EPDs) in growth, fertility and carcass traits, for use as selection criteria. The identification of relationships between molecular markers and EPDs by molecular analysis is an interesting approach for tracing genetic patterns among a wide range of economically important traits, and determining trends in the alleles of one in particular. The aim hereof was to analyze the relationship between the length of *MUC1* VNTRs and EPDs in Nelore beef cattle, in order to evaluate the effect of this polymorphism on economically important traits.

The sample consisted of 279 Nelore females from the same farm, the daughters of 37 elite sires. EPDs were calculated through the Program for Genetic Improvement of the Nelore Breed (PMGRN) at FMRP, University of Sao Paulo, Brazil (ANCP), which applies pedigree data from approximately 954,706 animals to obtain the EPDs for all these animals so as to increase accuracy in prediction. EPDs for the following traits were estimated: weight at 120 days, including direct (W_120_) and maternal effect (MW_120_), as growth traits; scrotal circumference at 365 (SC_365_) and 450 days (SC_450_), and age at first calving (AFC), as fertility traits, and rib eye area (REA) as a carcass trait. EPDs were estimated by the restricted maximum likelihood method using MTDFREML software.

Five mL of whole blood was collected from the caudal vein of each animal, direct into vacuum tubes containing 7.5 mg EDTA. Genomic DNA was extracted by precipitation in NaCl using standard techniques (Olerup and Zetterquist, 1992). The animals were genotyped for the *MUC1* gene by the polymerase chain reaction (PCR). Forward and reverse primer sequences (5-CGCAGA ACT ACG CCA GTT TCC-3' and 5'-AGA GCGGGT GGT CAT GGA TG-3) were based on the bovine sequence deposited in GenBank under accession number AF399757, and adapted from the primer sequences which flank the repetitive VNTR region of bovine *MUC1*, as published by Rasero *et al.* (2002). About 100 ng of genomic DNA was mixed with 15 pmol of each primer, to a total volume of 25 μL containing 200 μ]M of each dNTP, 0.75 mM MgSO_4_, 0.5 U Platinum Pfx Taq DNA polymerase, 1X Platinum Pfx Amplification Buffer, and 1X PCR Enhancer Solution (Invitrogen, Carlsbad, CA, USA). PCR was performed in a Whatman Biometra T-Gradient Thermocycler under the following conditions: a denaturation step at 95 °C for 5 min, 35 cycles at 95 °C for 40 s, 58 °C for 40 s, 68 °C for 1 min and 30 s, and a final extension step at 68 °C for 5 min. Amplified fragments were separated by electrophoresis on 1.5% agarose gels prepared with 0.5X Tris-borate-EDTA buffer. Gels were photographed and allele lengths estimated using Kodak Digital Science1D Image Analysis Software. Plus DNA Ladder (1 kb) was used as molecular weight standard.

The maternal and animal EPDs predicted for 279 females were independently compared with theirs genotypes by analysis of variance (ANOVA) using the general linear model (GLM) of the Statistical Analysis Software (SAS) (Statistical Analysis System, 2001). The following linear model was used: *y*_*ij*_ = μ + *G*_*i*_ + *e*_*ij*_, where *y*_*ij*_ is the EPD for each trait of the *ij*^th^ animal, μ is the general mean of each trait, *G*_*i*_ is the fixed effect of the *i*^th^*MUC1* VNTR genotype, and *e*_*ij*_ is the random effect associated with the *ij*^th^ observation.

Five alleles with different lengths were amplified and named, according to Souza *et al.* (2007), who first described these alleles in Nelore cattle. The model described by Souza *et al.* (2007) was used to estimate the number of repeats in each allele using allele 1036 as parameter, as this presented the same size as the 13-repeat allele described by Rasero *et al.* (2002). Allele 1175 contained 15 repeats, allele 1323 contained 18 , allele 1526 consisted of 21, and allele 1894 contained 27 repeats.

All alleles reported in this study were similar to those already described for *Bos primigenius indicus* (Souza *et al.*, 2007). Allele 1036 was the most frequent (frequency of 0.739) and allele 1894 was the least frequent (frequency of 0.007). Eleven genotypes were identified, with the combination of alleles with short VNTRs being significantly higher than the combination of long VNTRs. The allele and genotype frequencies of obtained *MUC1* gene polymorphism are shown in Table 1.

No correlation between *MUC1* polymorphism and EPDs (p = 0.05) was evident, on analyzing the three most frequent genotypes (1036/1036, 1036/1175 and 1036/1323). W_120_ was the trait that presented a value close to significance (p = 0.08). (Table 2) The other genotypes presented low frequencies, and thus no correlation with EPDs could be established (Table 1).

In conclusion, the statistical analyses showed that there was no significant association between *MUC1* VNTR length and EPDs for W120, SC365, SC450, AFC and REA. Nevertheless, the results obtained are in agreement with those of Souza *et al.* (2007), showing that Brazilian Nelore cattle are characterized by high frequencies of short VNTRs and low frequencies of long VNTRs of the *MUC1* gene.

## Figures and Tables

**Table 1:** Allele and genotype frequencies of *MUC1* gene polymorphism in 279 Nelore cattle.

Allele	Frequency	Genotype	Frequency
1036	0.793	*1036/1036*	0.670
1175	0.080	*1036/1175*	0.103
1323	0.087	*1036/1323*	0.100
1526	0.027	*1036/1526*	0.030
1894	0.007	*1036/1894*	0.003
		*1175/1175*	0.025
		*1175/1323*	0.007
		*1323/1323*	0.035
		*1323/1894*	0.003
		*1526/1526*	0.007
		*1894/1894*	0.003

**Table 2:** Least square means and standard deviation of growth, fertility and carcass expected progeny differences (EPD) obtained for the three most frequent genotypes of the *MUC1* gene, and the respective p values obtained by analysis of variance.

EPD	p (0.05)	*MUC1* genotype		
		1036/1036	*1036/1175*	*1036/1323*
W_120_ (kg)	0.08	3.68 ± 1.64	3.30 ± 1.85	3.16 ± 2.43
MW_120_ (kg)	0.29	0.90 ± 1.25	1.24 ± 0.77	1.06 ± 1.13
SC_365_ (cm)	0.37	9.62 ± 4.07	9.49 ± 3.85	8.93 ± 5.07
SC_450_ (cm)	0.79	10.0 ± 4.97	9.71 ± 4.62	9.40 ± 6.07
AFC (days)	0.72	-0.56 ± 0.49	-0.46 ± 0.37	-038 ± 0.39
REA (cm^2^)	0.82	0.49 ± 0.54	0.38 ± 0.55	0.44 ± 0.54

W_120_ and MW_120_: genetic direct and maternal effects for weight at 120 days, respectively; SC_365_ and SC_450_: scrotal circumference at 365 and 450 days; AFC: age at first calving; REA: rib eye area.
